# Long-Term Mortality after Transcatheter Edge-to-Edge Mitral Valve Repair Significantly Decreased over the Last Decade: Comparison between Initial and Current Experience from the MiTra Ulm Registry

**DOI:** 10.3390/jcm13082172

**Published:** 2024-04-10

**Authors:** Nicoleta Nita, Marijana Tadic, Johannes Mörike, Michael Paukovitsch, Dominik Felbel, Mirjam Keßler, Matthias Gröger, Leonhard-Moritz Schneider, Wolfgang Rottbauer

**Affiliations:** Department of Internal Medicine II, University Medical Center, 89081 Ulm, Germany; marijana.tadic@uniklinik-ulm.de (M.T.); johannes.moerike@uniklinik-ulm.de (J.M.); michael.paukovitsch@uniklinik-ulm.de (M.P.); dominik.felbel@uniklinik-ulm.de (D.F.); mirjam.kessler@uniklinik-ulm.de (M.K.); matthias.groeger@uniklinik-ulm.de (M.G.); leonhard-moritz.schneider@uniklinik-ulm.de (L.-M.S.); wolfgang.rottbauer@uniklinik-ulm.de (W.R.)

**Keywords:** M-TEER long-term outcome, survival trends after M-TEER, prospective register M-TEER

## Abstract

(1) **Objective**: We aimed to assess whether the candidate profile, the long-term outcomes and the predictors for long-term mortality after transcatheter edge-to-edge mitral valve repair (M-TEER) have changed over the last decade; (2) **Methods**: Long-term follow-up data (median time of 1202 days) including mortality, MACCE and functional status were available for 677 consecutive patients enrolled in the prospective MiTra Ulm registry from January 2010 to April 2019. The initial 340 patients treated in our institution before January 2016 were compared with the following 337 patients; (3) **Results**: Patients treated after 2016 showed significantly less ventricular dilatation (left ventricular end-systolic diameter of 43 ± 13 mm vs. 49 ± 16 mm, *p* < 0.007), lower systolic pulmonary pressures (50 ± 15 mmHg vs. 57 ± 21 mmHg, *p* = 0.01) and a lower prevalence of severe tricuspid regurgitation (27.2% vs. 47.3%, *p* < 0.001) at baseline than patients treated before 2016. Compared to the cohort treated before 2016, patients treated afterwards showed a significantly lower all-cause 3-year mortality (29.4% vs. 43.8%, *p* < 0.001) and lower MACCE (38.6% vs. 54.1%, *p* < 0.001), without differences for MR etiology. While severe tricuspid regurgitation and NYHA class IV remained independently associated with an increased long-term mortality over the last decade, severe left ventricular dilatation (hazard ratio, HR 2.12, *p* = 0.047) and severe pulmonary hypertension (HR 2.18, *p* = 0.047) were predictors of long-term mortality only in patients treated before 2016. (4) **Conclusions**: The M-TEER candidates are currently treated earlier in the course of disease and benefit significantly in terms of a better long-term survival than patients treated at the beginning of the M-TEER era.

## 1. Introduction

Over the past decade, transcatheter mitral edge-to-edge repair (M-TEER) has revolutionized the management of inoperable patients suffering from severe mitral regurgitation (MR). Improvements in heart failure symptoms, quality of life and short-term survival have been documented in several trials [1,2,3,4], leading to increasing recognition and acceptance of M-TEER in recent European and American guidelines for both primary and secondary MR [5].

However, discrepant data have been reported in recent years regarding long-term outcomes after the procedure, with long-term mortality rates at three to four years ranging from 17% in the Everest II trial to 53% in the German TRAMI registry [6,7]. The largest trials in this field, the COAPT trial and MITRA-FR trial, revealed divergent mid-term cardiovascular outcomes in functional MR; however, the latest long-term follow-up results of the COAPT trial showed that the survival benefit achieved at 24 months after the procedure persisted to 36 months [2,8,9,10].

The differing reports on survival reflect the differences in inclusion criteria, the management of comorbidities and the operator skill level [11]. Nevertheless, acknowledgment of these discrepancies should improve the patient selection, prognosis assessment and therapy timing in mitral regurgitation. Although multiple studies have published survival data, evidence on long-term outcomes is still scarce, and survival trends from real-world registries have not been published so far.

The aim of the present study was to determine whether the long-term outcomes including mortality and major adverse cardiac and cerebrovascular events (MACCE) after M-TEER at our institution have changed over the last decade and to determine the predictors of improved survival that may facilitate the future selection of optimal candidates who will benefit most from the procedure.

## 2. Materials and Methods

### 2.1. Study Population

The present study included 677 consecutive patients with symptomatic moderate-to-severe and severe MR who underwent transcatheter edge-to-edge mitral valve (MV) repair at our institution between January 2010 and April 2019. All patients were enrolled in the prospective MiTra Ulm registry and gave informed consent for the registry participation. There were no exclusion criteria for the enrolment in our prospective registry. Out of a total of 750 patients treated during this period, 73 patients were lost to follow-up. The following analyses are based only on patients for whom long-term follow-up data were available for at least 3 years. Of note, there were no relevant differences in baseline characteristics between the study patients and those lost to follow-up (Supplemental Table S1). Eligibility for the M-TEER procedure was determined by the multidisciplinary heart team according to valvular heart disease guidelines [12]. The MitraClip (Abbott) device was used for MV repair under fluoroscopic and echocardiographic guidance in a hybrid catheterization laboratory. Over the past decade, both the early generation (Clasic and NT) and the newer generation of the MitraClip device (NTR, 9 mm arm length and XTR with 12 mm extended arms) have been successfully implanted. The registry protocol was approved by the ethical committee of the University of Ulm and complied with the Declaration of Helsinki (NCT03104660/2011).

### 2.2. Outcomes and Definitions

Procedural and acute outcomes were assessed according to the recommendations of the Mitral Valve Academic Research Consortium (MVARC) [13]. In particular, the technical procedural success, number of clips deployed, device time, fluoroscopy time, post-procedural mean gradient and post-procedural MR grade, as well as the 30-day all-cause death, MACCE and rehospitalization, were reported. Long-term outcome data were evaluated at a 3-year follow-up and included functional clinical assessment based on New York Heart Association (NYHA) classification, all-cause and cardiovascular death and reintervention, as well as MACCE, which included death from any cause, stroke and myocardial infarction. Follow-up was by routine clinical visit or telephone contact after device implantation. In order to identify potential differences and trend changes in long-term outcomes over the last decade, we divided the study population chronologically into two subgroups: the first cohort included 340 patients treated at our institution before 2016, and the second cohort included 337 patients who underwent the procedure after January 2016.

### 2.3. Statistics

Categorical variables were expressed as absolute numbers and percentages and compared by an X or Fisher’s test. The normality of distribution of continuous variables was analyzed using the Kolmogorov–Smirnov and Shapiro–Wilk tests. Continuous variables were presented as the mean with standard deviation and compared using a Student’s *t*-test or Mann–Whitney–Wilcoxon test, respectively. Optimal cut-off values for systolic pulmonary pressures (sPAPs) and a left ventricular end-systolic diameter (LV-ESD) with an area under the curve (AUC) greater than 0.7 associated with an increased mortality risk were generated from the receiver operating characteristic (ROC) analysis using the Youden threshold.

Cumulative 3-year rates of all-cause mortality and MACCE were estimated using the Kaplan–Meier method, and the differences between initial and recently treated subgroups were calculated using the log-rank test. Multivariable Cox regression analysis using a stepwise forward selection was performed to assess the influence of relevant baseline and procedural variables on 3-year mortality and MACCE. The algorithm was applied to all potentially influential parameters (*p* < 0.10) from the univariate logistic regression analysis. Collinearity between parameters was analyzed using variance inflation factors. Predictors of long-term mortality were evaluated separately for the two subgroups in order to identify differences occurring over time. All tests were two-tailed, and a *p*-value < 0.05 was considered statistically significant. SPSS statistical package version 20.0 (SPSS Inc., Chicago, IL, USA) 9.3 (Cary, NC, USA) was used for calculations.

## 3. Results

### 3.1. Baseline Characteristics

Baseline characteristics of the study population and the differences between initially treated and recently treated patients are shown in Table 1. Of the 677 patients included in the analysis (76 ± 8 years, male sex: 59.4%, EuroSCORE II 8.4 ± 8.2%), functional MR (FMR) was the predominant etiology (*n* = 429/677, 63.3%). However, the prevalence of FMR significantly decreased over time from 68.4% in the initially treated cohort to 58.2% in the recently treated cohort (*p*-value < 0.001). The gender distribution of patients did not change significantly over the last decade. Concerning the severity of heart failure (HF) symptoms in the total population, NYHA class III was more prevalent than NYHA class IV. However, in the subgroup comparison, the proportion of patients with NYHA class IV treated before 2016 was significantly higher than in the cohort treated after 2016 (46.2% vs. 26.4%, *p* < 0.001, Table 1). The same trend was observed for the rate of prior decompensation. There was a high burden of comorbidities in the entire study population, including coronary artery disease (69.3%), atrial fibrillation (65.1%), previous cardiac surgery (21.9%), chronic kidney disease (53.9%) and diabetes mellitus (28.3%). No relevant differences in the comorbidity profile were observed between initially and recently treated patients. Baseline laboratory tests showed significant higher levels of cardiac markers such as troponin T and NT-pro BNP in the initially treated cohort than in the recently treated cohort (Supplemental Table S2). Similar results were observed with the baseline EuroScore II.

### 3.2. Echocardiographic and Invasive Hemodynamic Assessment

Echocardiographic evaluation at baseline revealed a mean left ventricular ejection fraction (LVEF) of 44 ± 17% with no significant differences between subgroups. Patients treated before 2016 had significantly more atrial and ventricular dilatation, significantly higher sPAP values and a higher proportion of severe tricuspid regurgitation than patients treated after 2016. Similar results were observed for the effective regurgitant orifice area (EROA), as shown in Table 2. The proportion of patients with complex degenerative valve anatomy (Carpentier type IIIa) increased significantly over the last decade (20.4% in the recently treated cohort vs. 12.3% in the initial cohort, *p* = 0.002), whereas, although statistically not significant, MR related to ring/atrial dilatation (Carpentier type I) was more prevalent in the initially treated cohort (Table 2). The hemodynamic parameters measured during cardiac catheterization prior to device implantation are shown in Table 2. Patients treated initially had significantly higher values for mean right ventricular (RV) pressures, systolic pulmonary artery (PA) pressures and mean and peak left atrial (LA) pressures than patients treated after 2016.

### 3.3. Short-Term Outcomes

Procedural and 30-day outcomes are reported in Table 3. No significant differences were observed between initially treated and recently treated patients regarding the post-procedural mitral regurgitation, the number of devices implanted and the post-procedural mean gradient. Moreover, the MVARC procedural success and short-term outcomes including mortality, rehospitalization and MACCE were similar between the two groups. Instead, the fluoroscopy time, post-procedural LA pressures and length of post-procedural hospitalization were significantly higher in patients treated initially than in patients treated more recently.

### 3.4. Functional Outcomes in the First vs. Current M-TEER Experience

**Figure 1:**
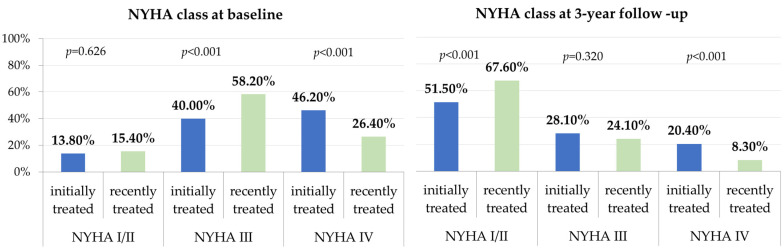
NYHA functional class at baseline and 3-year follow-up for initially vs recently treated patients. At baseline, the proportion of patients with NYHA class IV was significantly higher for patients treated before 2016. At 3-year follow-up, the percentage of patients with NYHA functional class I/II was significantly lower in the initially treated cohort than in the recently treated cohort.

In the entire cohort, significant improvements in NYHA functional class were observed at the 1-year follow-up and maintained during the long-term follow-up (NYHA I/II in 63.3% at 3-year follow-up vs. 69.5% at 1-year follow-up vs. 14.7% before the procedure). However, in the subgroup analysis, the percentage of patients with NYHA functional class I/II at the 3-year follow-up was significantly lower in the initially treated cohort than in the recently treated cohort (51.5% vs. 67.6%, *p* value < 0.001). In addition, the proportion of patients with NYHA class IV at the 1-year and 3-year follow-ups was significantly higher in the cohort treated before 2016 than in the subgroup treated after 2016 (16.9% vs. 8.1%, *p* < 0.001 at 1-year follow-up and 20.4% vs. 8.3%, *p* < 0.001 at 3-year follow-up), as shown in Figure 1.

### 3.5. Trends in Long-Term Follow-Up over the Last Decade

**Figure 2:**
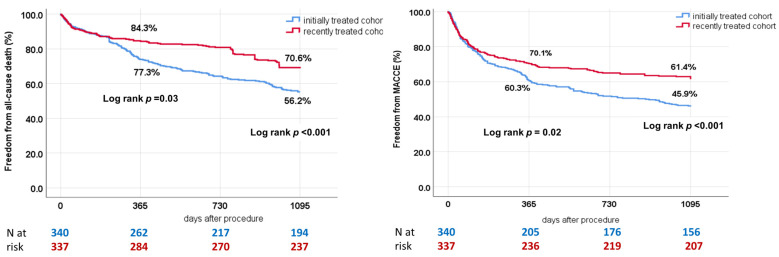
Kaplan–Meier curves for mortality and MACCE for initially vs recently treated patients. Recently treated patients have significantly lower mortality and MACCE rates than patients treated before 2016.

Mortality rates in the total cohort, as estimated by the Kaplan–Meier method, were 19.1% at the 1-year follow-up and 36.6% at the 3-year follow-up. Recently treated patients had a significantly higher overall survival rate compared to patients treated in the first experience at both the 1-year and 3-year follow-ups. The same was observed for MACCE rates, as shown in Figure 2.

**Figure 3:**
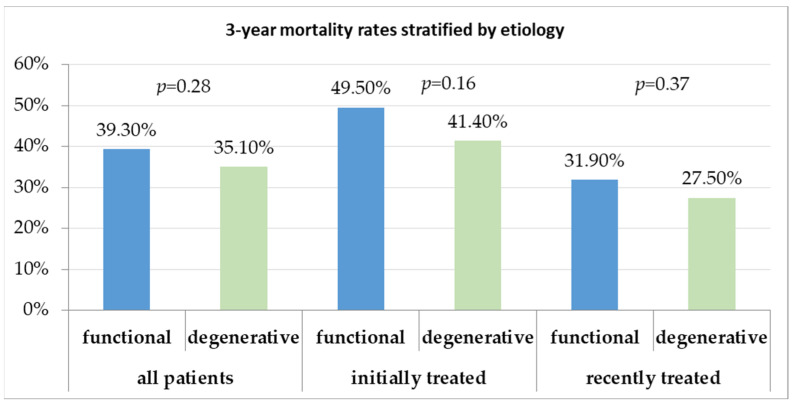
Long-term mortality rates stratified according to etiology. No significant differences for long-term survival between functional and degenerative mitral regurgitation were observed.

Stratification for MR etiology did not show significant differences for long-term survival either in the whole study population (proportion of all-cause 3-year death in functional MR and FMR was 39.3% compared to 35.1% in degenerative MR and DMR, *p* = 0.28) or in the subgroup analysis (proportion of all-cause death in the initially treated cohort in FMR was 49.5% vs. 41.4% for DMR, *p* = 0.163; proportion of all-cause death in the recently treated cohort in FMR was 31.9% vs. 27.5% for DMR, *p* = 0.37, Figure 3). All-cause mortality was the predominant adverse event during the long-term follow-up, with cardiovascular causes accounting for 67% of all deaths. During long-term follow-up, percutaneous mitral valve reintervention was required in 6.4% patients, with more procedures required in initially treated patients than in patients treated more recently (8% vs. 4.6%, *p* = 0.057). There was no significant difference between FMR and DMR regarding the reintervention rate (5.7% vs. 7.3%, *p* = 0.366).

### 3.6. Predictors of Long-Term Mortality over the Last Decade

To determine differences in predictors of long-term mortality over the past decade, baseline and procedural factors were compared between survivors and non-survivors at the 3-year follow-up separately for initially and currently treated patients. Variables that were statistically significant in univariable analysis were further subjected to multivariable Cox regression analysis. In order to avoid multicollinearity and redundancy, the Euroscore II, which was predictive of long-term mortality in both subgroups in the univariate analysis, was not included in the multivariate model.

**Figure 4:**
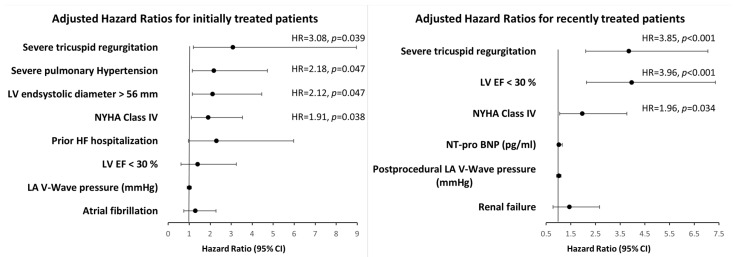
Hazard ratios for various variables as predictors of all-cause death for initially vs. recently treated patients. Severe pulmonary hypertension is defined as systolic pulmonary artery pressure > 60 mmHg; HF, heart failure; LA, left atrial; LV, left ventricular; EF, ejection fraction.

For the cohort treated after 2016, multivariable Cox regression analysis identified baseline severe tricuspid regurgitation (HR 3.85, *p* < 0.001), LVEF < 30% (HR 3.96, *p* < 0.001) and baseline NYHA class IV (HR 1.96, *p* = 0.034) as relevant independent predictors for long-term mortality, as depicted in Figure 4 and Supplemental Table S3. In contrast, multivariable Cox regression analysis performed for patients treated initially identified, in addition to severe tricuspid regurgitation and baseline NYHA class IV, severe pulmonary hypertension (defined as sPAP > 60 mmHg, cut-off value generated from the ROC analysis, HR 2.18, *p* = 0.047) and severe LV dilatation (LVESD > 56 mm, cut-off value generated from the ROC analysis, HR 2.12, *p* = 0.047) as independent predictors of long-term mortality (Figure 4).

## 4. Discussion

The MiTra Ulm registry represents a large cohort of real-world patients treated with transcatheter edge-to-edge mitral valve repair. This study reports on trends in long-term outcomes during a 10-year M-TEER experience and revealed several important findings that merit further discussion: (i) the patient profile has changed significantly over the last decade in terms of baseline cardiac remodeling, hemodynamic status, medical therapy and the complexity of valve morphology; (ii) the percentage of patients with NYHA functional class I/II at the 3-year follow-up was significantly lower in the initially treated cohort than in the cohort treated after 2016; (iii) although post-procedural LA pressures and the length of post-procedural hospitalization were significantly higher in patients treated early in the M-TEER era, no significant differences were observed between initially and recently treated patients in short-term outcomes including mortality, rehospitalization and MACCE rates; (iv) recently treated patients had significantly lower mortality and MACCE rates compared with patients treated in the initial experience at both 1-year and 3-year follow-ups; (v) stratification by MR etiology showed no significant differences in long-term survival for either initially treated patients or patients treated after 2016; (vi) severe pulmonary hypertension and ventricular dilatation predicted long-term mortality only in the initially treated cohort.

The baseline demographics and comorbidity burden of the prospective MitraUlm cohort are similar to those that have been already published by other European registries [3,7,11,14,15] and have not changed significantly over the last decade.

However, in contrast to the TRAMI registry, which reported a tendency to treat sicker patients with higher stages of heart failure [3,7], we observed a significant decrease in terminal heart failure symptoms in recent years, with the proportion of patients with NYHA class IV at baseline decreasing from 58.8% to 40.9%. These clinical findings correlate with the extent of cardiac adverse remodeling, as patients nowadays present with less atrial and ventricular dilatation. Accordingly, the proportion of severe concomitant tricuspid regurgitation decreased significantly from 47.3% to 27.3%. The pre-procedural hemodynamic assessment supports these findings, as patients currently treated have lower LA pressures, lower right heart pressures and a significantly lower proportion of severe pulmonary hypertension. All of these clinical, echocardiographic and hemodynamic findings suggest that patients are now being treated earlier in the course of their disease, before irreversible cardiac remodeling occurs.

The current analysis reports, in agreement with other real-world registry data, a significant overall improvement in functional outcomes at the 1-year follow-up, which persisted through long-term follow-up. Yet, we found that more recently treated patients had better symptomatic alleviation at the 3-year follow-up than patients treated initially. This trend in clinical outcomes seems to correlate with the aforementioned change in the baseline patient profile and with the improved medical therapy in recent years.

Acute changes in left atrial pressure after M-TEER have been correlated with the procedural success and clinical improvement [16]. We found that recently treated patients had significantly lower post-procedural LA pressures than patients treated initially. However, these hemodynamic changes have not had an immediate impact on short-term outcomes, as 30-day mortality, rehospitalization and MACCE rates have not changed significantly over the last decade.

The present study reports a mortality rate of 36% at the 3-year follow-up, which is similar to other registries but slightly lower than data from the German TRAMI registry [7].

The most important finding of the current analysis relates to the fact that mortality rates have decreased significantly over the last decade, with long-term death rates dropping from 44% in the first 5 years of M-TEER experience to 29% for the patients treated after 2016. These trends are even more evident for the 3-year MACCE rates, which improved from 54% in the beginning to 38% nowadays. The rate of percutaneous MV reintervention in the whole cohort was low, in accordance with previous reports from the EVEREST II trial or the TRAMI registry [7,14]. However, patients treated more recently in our MiTra Ulm registry showed significantly lower rates of reintervention.

Interestingly, stratification by MR etiology showed no significant differences in long-term survival. This has not changed over the last decade, although the proportion of patients with degenerative MR has increased significantly over time. The rising prevalence of degenerative MR is not due to an increase in the proportion of Carpentier Type II in patients with normal LV function but rather to an increase in the number of patients with complex degenerative valve anatomy being treated at our institution. This, however, reflects the higher level of expertise that has been accumulated over the past decade. Although not statistically significant, the proportion of patients with functional MR due to atrial/ring dilatation was higher in the initially treated cohort, which correlates with the extensive cardiac dilatation observed in the first cohort. To our knowledge, only one registry-based study has previously compared long-term survival between etiologies before, namely the long-term follow-up study from the TRAMI registry, which reported similar results to ours and suggested that mortality in this elderly population is mainly driven by the multimorbidity and not by etiology [7].

In order to provide a proper explanation for the improved long-term survival over time, we analyzed the predictors for mortality separately for patients treated between 2010 and 2016 and between 2016 and 2019. Regarding the baseline clinical, echocardiographic and hemodynamic profile, we observed that the M-TEER candidate currently presents significantly lower cardiac dilatation, a lower prevalence of severe pulmonary hypertension and lower left atrial pressures, despite their advanced age and high comorbidity burden. Furthermore, the proportion of patients with severe heart failure symptoms at baseline (NYHA IV stage) and the prevalence of prior decompensation have significantly decreased over the last decade. This improvement in clinical status before the procedure can be attributed to the documented advancements in heart failure medical therapy.

The multivariate Cox regression analysis confirmed these observations and identified cardiac adverse remodeling and severe pulmonary hypertension as independent predictors for long-term mortality only in the subgroup of patients treated initially. Notably, these predictors of mortality overlap with the exclusion criteria of the COAPT trial, which demonstrated a clear survival benefit in patients without severe cardiac dilation and severe pulmonary hypertension [17,18,19]. Nevertheless, severe tricuspid regurgitation, although significantly less prevalent in recent years, remained a relevant independent predictor of mortality during the last decade.

### Limitations

Several limitations of this study require further consideration. Firstly, the results are based on our local registry data, and although our patients were consecutively enrolled, the possibility of under-reporting of adverse events cannot be excluded. Secondly, baseline echocardiographic data were not core-lab adjudicated, which could affect the results due to unstandardized measurements. Being a single-center study, the findings cannot be easily generalized. Furthermore, the long-term follow-up data, particularly the functional clinical data, were incomplete (90%), which carries an inherent risk of selection bias. To address this limitation, we compared patients with and without follow-up and found no significant differences in their baseline characteristics. Registries cannot replace randomized results; however, our large cohort of real-life patients treated over a decade offers a valuable opportunity to observe the evolution of M-TEER over time. Patients with previous cardiac valvular surgery were included in the analysis, which may have influenced the outcome data. However, this was a real-world multimorbid population of patients treated for severe mitral regurgitation, and we consider the inclusion of all patients to be a relevant strength of the current study. Our study did not include patients treated during the COVID-19 pandemic. Delayed treatment during the pandemic may have disrupted the positive evolution regarding the long-term outcomes observed in our study. Future studies should be conducted in order to determine whether the improved outcomes observed in recently treated patients also apply to patients treated during and after the pandemic.

## 5. Conclusions

The current analysis is based on a large cohort of patients with severe MR who underwent M-TEER at our institution during the last decade. The profile of M-TEER candidates has changed over the last 10 years, with less adverse cardiac remodeling and a better hemodynamic status despite more complex degenerative valve morphology. Long-term mortality and MACCE rates have decreased significantly over time. Different predictors for long-term mortality have been identified: while severe tricuspid regurgitation and NYHA class IV at baseline remained independently associated with an increased long-term mortality over the past decade, severe left ventricular dilatation and severe pulmonary hypertension predicted long-term mortality only in patients treated in the early M-TEER era. Although current guidelines recommend the procedure only for patients who remain symptomatic despite optimal medical therapy, our results suggest that timely treated patients benefit most from M-TEER before the onset of irreversible cardiac remodeling and severe pulmonary hypertension.

## Figures and Tables

**Table 1:** Baseline characteristics of study population.

	All (*n* = 677)	Initially Treated (*n* = 340)	Recently Treated (*n* = 337)	*p*-Value
Age (years)	76 ± 8	76 ± 8	76 ± 9	0.683
Male (%)	402 (59.4)	208 (61.1)	194 (57.6)	0.342
Functional MR (%)	429 (63.3)	233 (68.4)	196 (58.2)	0.004
NYHA class				
I–II (%)	99 (14.7)	47 (13.8)	52 (15.4)	0.626
III (%)	332 (49.0)	136 (40.0)	196 (58.2)	<0.001
IV (%)	246 (36.3)	157(46.2)	89 (26.4)	<0.001
Prior heart failure hospitalization	338 (49.9)	200 (58.8)	138 (40.9)	<0.001
Interventions and surgeries				
PCI (%)	308 (45.5)	143(42.1)	165 (48.9)	0.060
CABG (%)	108 (16)	60 (17.7)	48 (14.2)	0.273
Valve surgery (%)	40 (5.9)	22 (6.4)	18 (5.3)	0.566
Comorbidities				
Previous MI (%)	159 (23.5)	70 (20.6)	89 (26.4)	0.103
CAD (%)	469 (69.3)	246 (72.5)	223 (66.1)	0.064
DCM (%)	123 (18.2)	59(17.4)	64(18.9)	0.605
Hypertension (%)	544 (80.4)	280 (82.3)	264 (78.3)	0.185
Diabetes (%)	192 (28.3)	112 (33)	80 (23.7)	0.177
Atrial fibrillation (%)	441 (65.1)	228 (67.1)	213 (63.2)	0.273
Peripheral artery disease (%)	60 (8.9)	31 (9.4)	29 (8.6)	0.676
COPD (%)	84 (12.4)	47 (13.7)	37 (10.9)	0.299
Chronic renal failure (%)	365 (53.9)	190 (56)	175 (52)	0.252
Previous cancer (%)	116 (17.2)	61 (18)	55 (16.3)	0.203
Antiarrhythmia devices				
CRT (%)	66 (9.8)	36 (10.7)	30 (8.9)	0.369
ICD (%)	97 (14.3)	68 (20)	29 (8.6)	0.022
Pacemaker (%)	62 (9.2)	36 (9.9)	26 (8.7)	0.505
Euro Score II	8.4 ± 8.2	9.6 ± 8.1	7.8 ± 8.2	<0.001
Therapy				
Loop diuretics (%)	523 (77.3)	264 (77.7)	259 (76.8)	0.757
ACEI (%)	319 (47.1)	173 (51)	146 (43)	0.034
ARB (%)	185 (27.3)	82 (24.1)	103 (31)	0.037
ARNI (%)	78(11.5)	8(2.3)	70 (20.7)	0.001
Beta-blockers (%)	586 (86.6)	292 (86)	294 (87.2)	0.501
Aldosterone antagonists (%)	304 (44.9)	121 (41)	183 (54)	0.032
Laboratory				
GFR (mL/min/1.73 m^2^)	47 ± 19	46 ± 18	49 ± 20	0.137
Troponin T (ng/L)	87 ± 144	139 ± 211	43 ± 98	0.021
NT-pro-BNP (pg/mL)	6601 ± 7238	7264 ± 7011	5995 ± 7398	0.001

ACEI—angiotensin-converting enzyme inhibitor, ARB—angiotensin II receptor blocker, ARNI—angiotensin receptor II blocker—neprilysin inhibitor, CABG—coronary artery bypass grafting, COPD—chronic obstructive pulmonary disease, CRT—cardiac resynchronization therapy, GFR—glomerular filtration rate, ICD—implantable cardiac defibrillators, MI—myocardial infarction, PCI—percutaneous coronary artery intervention.

**Table 2:** Echocardiographic and hemodynamic data of study population.

	All (*n* = 677)	Initially Treated (*n* = 340)	Recently Treated (*n* = 337)	*p*-Value
Echocardiography				
LVEF (%)	44 ± 17	43 ± 17	44 ± 17	0.434
LVEDD (mm)	60 ± 11	62 ± 12	58 ± 10	0.017
LVESD (mm)	47 ± 15	49 ± 16	43 ± 13	0.007
Interventricular septum thickness (mm)	11 ± 4.8	10.5 ± 2.4	11.3 ± 6	0.039
LA (mm)	57 ± 10	58 ± 12	55 ± 8	0.045
sPAP	59 ± 17	62 ± 20	57 ± 15	0.047
EROA	0.43 ± 0.35	0.48 ± 0.4	0.4 ± 0.31	0.003
Carpentier Type I	205(30.1)	111(32.7)	94(27.6)	0.139
Carpentier Type II	141(20.9)	67(19.7)	74(21.7)	0.553
Carpentier Type IIIa	109(16.3)	42(12.3)	67(20.4)	0.002
Carpentier Type IIIb	222(32.7)	120(35.3)	102(30.3)	0.147
Severe TR (grade III/IV)	253(37.3)	161(47.3)	92 (27.3)	<0.001
Catheterization				
Heart rate (beat/min)	72 ± 15	72 ± 14	71 ± 17	0.241
Mean RA pressure (mmHg)	11 ± 7	11 ± 6	10 ± 7	0.102
Mean RV pressure (mmHg)	26 ± 28	32 ± 32	13 ± 8	<0.001
Systolic PA pressure (mmHg)	55 ± 19	57 ± 21	50 ± 15	0.011
Diastolic PA pressure (mmHg)	23 ± 14	25 ± 15	19 ± 8	0.041
Mean PA pressure (mmHg)	34 ± 14	35 ± 15	32 ± 11	0.015
Mean LA pressure (mmHg)	27± 18	28 ± 18	22 ± 18	0.016
V wave LA pressure	32± 16	37 ± 18	30 ± 15	0.003
SVR (dynes/seconds/cm^−5^)	2019 ± 2201	2812 ± 529	1996 ± 2227	0.009
Cardiac index (L/min/m^2^)	2.08 ± 0.55	2.06 ± 0.57	2.13 ± 0.50	0.143

EROA—effective regurgitant orifice area, LA—left atrium, LV—left ventricle, LVEF—left ventricular ejection fraction, LVEDD—left ventricular end-diastolic diameter, LVESD—left ventricular end-systolic diameter, sPAP—systolic pulmonary artery pressure, PA—pulmonary artery, SVR—systemic vascular resistance, RA—right atrium, RV—right ventricle.

**Table 3:** Procedural and 30-day outcomes in the study population.

	All (*n* = 677)	Initially Treated (*n* = 340)	Recently Treated (*n* = 337)	*p*-Value
Time after procedure (days)	6.7 ± 5.6	7.69 ± 5.6	5.7 ± 5.3	<0.001
ICU length (days)	1.0 ± 3.9	1.05 ± 3.9	0.94 ± 3.9	<0.001
Fluoroscopy time (min)	29 ± 17	30 ± 17	27 ± 17	<0.001
Number of clips implanted	1.3 ±0.7	1.3 ±0.5	1.41 ±0.9	0.281
MVARC device success	635 (93.8)	317 (93.3)	318 (94.3)	0.660
Post-procedural mean LA pressure (mmHg)	15 ± 6	16 ± 6	12 ± 4	<0.001
Post-procedural v wave LA pressure	22 ± 11	24 ± 9	19 ± 11	<0.001
Grade of MR after procedure	1.5 ± 0.7	1.6 ± 0.7	1.5 ± 0.6	0.093
Transmitral mean gradient after procedure	3.4 ± 1.90	3.4 ± 2.2	3.5 ± 1.7	0.224
Periprocedural mortality	21 (3.1)	10 (2.9)	11 (3.2)	0.848
30-day rehospitalization	26 (3.9)	13 (3.8)	13 (3.9)	0.920
30-day all-cause mortality	27 (4)	12 (3.5)	15 (4.5)	0.470
30-day MACCE	51 (7.6)	27 (8)	24 (7.1)	0.703

ICU—intermediate care unit, LA—left atrium, MACCE—major adverse cardiac and cerebrovascular events, MR—mitral regurgitation, MVARC—Mitral Valve Academic Research Consortium.

## Data Availability

The datasets used and/or analyzed during the current study are available from the corresponding authors on reasonable request.

## Supplementary Materials

The following supporting information can be downloaded at https://www.mdpi.com/article/10.3390/jcm13082172/s1, Table S1: Baseline characteristics of study population vs. lost to follow-up; Table S2: Baseline laboratory values of study population; Table S3 Multivariable predictors for all-cause mortality (Cox regression model).
