# Supplementary material for: Long-Term Mortality after Transcatheter Edge-to-Edge Mitral Valve Repair Significantly Decreased over the Last Decade: Comparison between Initial and Current Experience from the MiTra Ulm Registry
Source: J Clin Med. 2024 Apr 10;13(8):2172. doi: 10.3390/jcm13082172 (PMC11050104; doi:10.3390/jcm13082172)
Supplement: Supplementary file 1 [file jcm-13-02172-s001.zip › jcm-2935621-supplementary.pdf]

Table S1. Baseline characteristics of study population vs. lost to follow-up.

	Included in study (n=677)	Lost to follow-up (n=73)	p-value
**Age (years)**	76 ± 8	78 ± 7	0.166
**Male (%)**	402 (59.4)	44 (60.3)	0.875
**Functional MR (%)**	401 (59.3)	39 (53.4)	0.137
**NYHA class**			
I-II (%)	99(14.6)	9(12.3)	0.574
III (%)	332 (49.0)	31 (42.4)	0.148
IV (%)	246(36.3)	32 (43.8)	0.086
**Prior heart failure hospitalization**	338 (49.9)	36 (49.3)	0.891
**Prior interventions**			
PCI (%)	308(45.5)	31 (42.5)	0.580
CABG (%)	108 (16)	15 (20.5)	0.515
**Comorbidities**			
CAD (%)	469 (69.3)	52 (71.2)	0.711
DCM (%)	123(18.26)	11(15.1)	0.471
Hypertension (%)	544 (80.4)	59 (80.8)	0.918
Diabetes (%)	192(28.3)	24 (32.9)	0.372
Atrial fibrillation (%)	441 (65.1)	49 (67.1)	0.717
COPD (%)	84 (12.4)	9 (12.3)	0.981
Chronic renal failure (%)	365 (53.4)	35 (47.9)	0.280
CRT (%)	66 (9.7)	4 (5.5)	0.196
**Euro Score II**	8.4 ± 8.2	7.8 ± 8.2	0.755
**Therapy**			
ACEI (%)	319 (47.1)	33 (45.2)	0.738
ARB (%)	185 (27.3)	17 (23.3)	0.421
ARNI (%)	78 (11.5)	7 (9.5)	0.482
Beta-blockers (%)	586 (86.7)	63(86.3)	0.931
Aldosterone antagonists (%)	304 (44.9)	26 (35.6)	0.083
**Laboratory**			
Troponin T (ng/l)	87 ± 144	111 ± 153	0.123
NT-pro-BNP (pg/ml)	6601 ± 7238	6595 ± 7398	0.698
**Echocardiography**			
LVEF (%)	44±17	48±15	0.082
LVESD (mm)	47±15	48±17	0.654
sPAP (mmHg)	59±17	62±18	0.446
Severe TR	253(37.3)	31(43.1)	0.288

ACEI – angiotensin converting enzyme inhibitor, ARB- angiotensin II receptor blocker, ARNI - angiotensin receptor II Blocker - neprilysin inhibitor, CABG – coronary artery bypass grafting, COPD – chronic obstructive pulmonary disease, CRT – cardiac resynchronization therapy, GFR – glomerular filtration rate, ICD – implantable cardiac defibrillators, , MI – myocardial infarction, PCI – percutaneous coronary artery intervention;.

Table S2. Baseline laboratory values of study population.

	Initially treated (n=340)	Recently treated (n=337)	p-value
GFR (ml/min/1.73m2)	46 ± 18	49 ± 20	0.137
Creatinine (µmol/l)	132±68	130±71	0.745
Troponin T (ng/l)	139 ± 211	43 ± 98	0.021
NT-pro-BNP (pg/ml)	7264 ± 7011	5995 ± 7398	0.001
INR	1.3±0.5	1.4±0.6	0.537
PTT (s)	39±22	37±12	0.165
Hb(g/dl)	12.9±3.1	12.2±1.9	0.174
Platelates (Giga/L)	198±85	197±75	0.869

Table S3. Multivariable predictors for all-cause mortality (Cox regression model).

Predictors of 3-yr all-cause death for patients treated between 2010-2016	HR	95%-CI	P Value
Severe tricuspid regurgitation	3.08	1.05-8.97	0.039
Severe pulmonary hypertension (s-PAP> 60 mmHg)	2.18	1.09-4.72	0.047
LV end systolic diameter > 56 mm	2.12	1.01-4.46	0.047
NYHA class IV	1.91	1.03-3.54	0.038
Prior heart failure hospitalization	2.30	0.97-5.47	0.057
LV ejection fraction <30%	1.41	0.61-3.24	0.416
LA v wave pressure	1.01	0.99-1.02	0.160
Atrial fibrillation	1.30	0.74-2.28	0.351
**Predictors of 3-yr all-cause death for patients treated between 2016-2019**	**HR**	**95%-CI**	**P Value**
Severe tricuspid regurgitation	3.85	2.10-7.05	<0.001
LV EF < 30%	3.96	2.13-7.35	<0.001
NYHA class IV	1.96	1.05-3.77	0.034
NT-pro BNP	1.01	0.95-1	0.223
Postprocedural v-wave LA pressure	1.02	0.99-1.05	0.105
Renal failure	1.44	0.78-2.66	0.236

LA, left atrium, LV, left ventricle; s-PAP, systolic pulmonary artery pressure;.
